# 3-Phenyl-4-{3-[(*p*-tol­yloxy)meth­yl]-7*H*-1,2,4-triazolo[3,4-*b*][1,3,4]thia­diazin-6-yl}sydnone

**DOI:** 10.1107/S160053681002982X

**Published:** 2010-07-31

**Authors:** Jia Hao Goh, Hoong-Kun Fun, Nithinchandra, B. Kalluraya

**Affiliations:** a X-ray Crystallography Unit, School of Physics, Universiti Sains Malaysia, 11800 USM, Penang, Malaysia; b Department of Studies in Chemistry, Mangalore University, Mangalagangotri, Mangalore 574 199, India

## Abstract

In the title compound (systematic name: 3-phenyl-4-{3-[(*p*-tol­yloxy)meth­yl]-7*H*-1,2,4-triazolo[3,4-*b*][1,3,4]thia­diazin-6-yl}-1,2,3-oxadiazol-3-ium-5-olate), C_20_H_16_N_6_O_3_S, an intra­molecular C—H⋯O hydrogen bond generates an *S*(6) ring motif. The 3,6-dihydro-1,3,4-thia­diazine ring adopts a twist-boat conformation. The 1,2,3-oxadiazole and 1,2,4-triazole rings are inclined to each other at an inter­planar angle of 44.13 (13)°. The phenyl ring makes an inter­planar angle of 67.40 (13)° with the attached 1,2,3-oxadiazole ring. In the crystal structure, adjacent mol­ecules are inter­connected into two-mol­ecule-thick arrays parallel to (100) *via* C—H⋯O and C—H⋯N hydrogen bonds. A short S⋯O contact [2.9512 (18) Å] is observed.

## Related literature

For general background to, and applications of materials related to the title compound, see: Hedge *et al.* (2008 ▶), Kalluraya & Rahiman (1997 ▶); Kalluraya *et al.* (2003 ▶). For graph-set descriptions of hydrogen-bond ring motifs, see: Bernstein *et al.* (1995 ▶). For related structures, see: Goh *et al.* (2010**a* ▶,*b* ▶,c* ▶). For the stability of the temperature controller used in the data collection, see: Cosier & Glazer (1986 ▶). For puckering parameters, see: Cremer & Pople (1975 ▶).
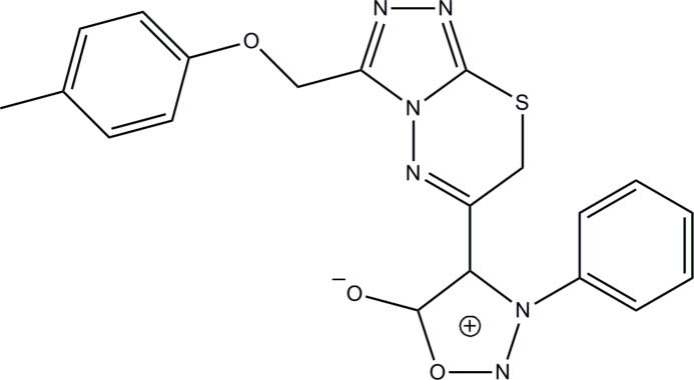


## Experimental

### 

#### Crystal data

C_20_H_16_N_6_O_3_S
*M* *_r_* = 420.45
Monoclinic, 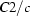

*a* = 42.0781 (12) Å
*b* = 8.2304 (2) Å
*c* = 11.1488 (3) Å
β = 101.630 (2)°
*V* = 3781.78 (17) Å^3^
*Z* = 8
Mo *K*α radiation
μ = 0.21 mm^−1^
*T* = 100 K
0.29 × 0.13 × 0.05 mm

#### Data collection

Bruker SMART APEXII CCD diffractometer
Absorption correction: multi-scan (*SADABS*; Bruker, 2009 ▶) *T*_min_ = 0.942, *T*_max_ = 0.989
11383 measured reflections
3486 independent reflections
2496 reflections with *I* > 2σ(*I*)
*R*_int_ = 0.059

#### Refinement

*R*[*F* ^2^ > 2σ(*F* ^2^)] = 0.051
*wR*(*F* ^2^) = 0.101
*S* = 1.03
3486 reflections
272 parameters
H-atom parameters constrained
Δρ_max_ = 0.38 e Å^−3^
Δρ_min_ = −0.40 e Å^−3^

### 

Data collection: *APEX2* (Bruker, 2009 ▶); cell refinement: *SAINT* (Bruker, 2009 ▶); data reduction: *SAINT*; program(s) used to solve structure: *SHELXTL* (Sheldrick, 2008 ▶); program(s) used to refine structure: *SHELXTL*; molecular graphics: *SHELXTL*; software used to prepare material for publication: *SHELXTL* and *PLATON* (Spek, 2009 ▶).

## Supplementary Material

Crystal structure: contains datablocks global, I. DOI: 10.1107/S160053681002982X/hb5565sup1.cif

Structure factors: contains datablocks I. DOI: 10.1107/S160053681002982X/hb5565Isup2.hkl

Additional supplementary materials:  crystallographic information; 3D view; checkCIF report

## Figures and Tables

**Table 1:** Hydrogen-bond geometry (Å, °)

*D*—H⋯*A*	*D*—H	H⋯*A*	*D*⋯*A*	*D*—H⋯*A*
C10—H10*A*⋯O3	0.97	2.27	3.041 (3)	135
C10—H10*A*⋯O3^i^	0.97	2.54	3.162 (3)	122
C10—H10*B*⋯O3^ii^	0.97	2.46	3.292 (3)	144
C19—H19*A*⋯N5^iii^	0.93	2.57	3.386 (3)	147

Symmetry codes: (i) 
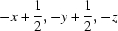
; (ii) 
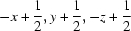
; (iii) 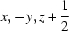.

**Fig. 1.**
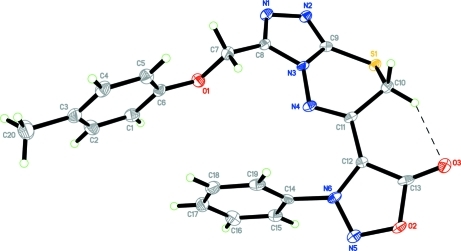
The molecular structure of (I), showing 50% probability displacement ellipsoids for non-H atoms. An intramolecular hydrogen bond is shown as dashed line.

**Fig. 2.**
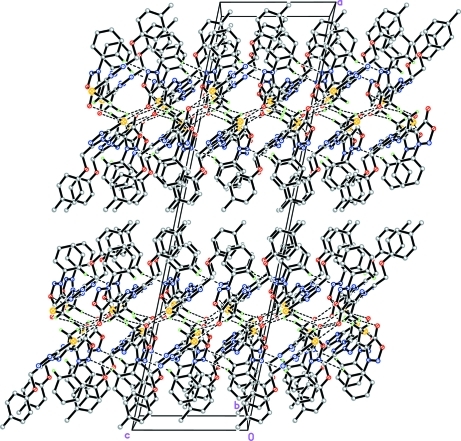
The crystal structure of (I), viewed along the b axis, showing two-molecule-thick arrays parallel to the (100) plane. Hydrogen atoms not involved in intermolecular interactions (dashed lines) have been omitted for clarity.

## supplementary crystallographic information

### Comment

Triazolothiadiazines have shown to possess significant biological and pharmacological activities such as anthelmintic, analgesic and anti-inflammatory (Kalluraya & Rahiman, 1997) properties. Encouraged by these literatures, we have synthesized triazolothiadiazines containing the sydnone moiety. The introduction of sydnone moiety into an heterocyclic compound will increase the biological and pharmacological activities of heterocyclic system (Hedge *et al.*, 2008). Triazolothiadiazines were synthesized by the condensation of 4-bromoacetyl-3-arylsydnones with 3-aryloxymethyl-4-amino-5-mercapto-1,2,4-triazoles. 4-Bromoacetyl-3-arylsydnones were in turn obtained by the photochemical bromination of 4-acetyl-3-arylsydnones (Kalluraya *et al.*, 2003).

In the title compound, (I), an intramolecular C10—H10A···O3 hydrogen bond (Table 1) generates a six-membered ring, producing an *S*(6) hydrogen bond ring motif (Fig. 1, Bernstein *et al.*, 1995). The 3,6-dihydro-1,3,4-thiadiazine ring (C9-C11/N3/N4/S1) adopts twist-boat conformation, with puckering parameters of Q = 0.630 (2) Å, θ = 67.03 (18)° and φ = 323.0 (2)° (Cremer & Pople, 1975). The essentially planar 1,2,3-oxadiazole (C12/C13/O2/N5/N6) and 1,2,4-triazole (C8/N1/N2/C9/N3) rings are inclined to each other at interplanar angle of 44.13 (13)°. The C14-C19 phenyl ring is inclined at interplanar angle of 67.40 (13)° with respect to the attached 1,2,3-oxadiazole ring. The geometric parameters are comparable to those reported in closely related structures (Goh *et al.*, 2010**a*,*b*,c*).

In the crystal structure, intermolecular C10—H10A···O3, C10—H10B···O3 and C19—H19A···N5 hydrogen bonds (Table 1) link adjacent molecules into two-molecule-thick arrays parallel to (100) plane (Fig. 2). Interestingly, further stabilization of the crystal structure is provided by intermolecular short S1···O3 interaction [2.9512 (18) Å; symmetry code: -x+1/2, -y+1/2, -z] which is significantly shorter than the sum of Van der Waals radii of the relevant atoms.

### Experimental

A solution of triazole (0.01 mol) and 4-bromoacetyl-3-phenylsydnone (0.01 mol) in absolute ethanol (20 ml) was heated under reflux for 10–12 h. The solution was concentrated, cooled to room temperature and neutrallized with 10 % sodium bicarbonate solution. The separated solid was filtered, washed with water, dried and recrystallized from ethanol. Colourless blocks of (I) were obtained from a 1:2 mixture of DMF and ethanol by slow evaporation.

### Refinement

All hydrogen atoms were placed in their calculated positions, with C—H = 0.93–0.97 Å, and refined using a riding model, with *U*_iso_ = 1.2 or 1.5 *U*_eq_(C). The rotating group model was used for the methyl group.

### Crystal data

**Table:**

C_20_H_16_N_6_O_3_S	*F*(000) = 1744
*M**_r_* = 420.45	*D*_x_ = 1.477 Mg m^−^^3^
Monoclinic, *C*2/*c*	Mo *K*α radiation, λ = 0.71073 Å
Hall symbol: -C 2yc	Cell parameters from 2771 reflections
*a* = 42.0781 (12) Å	θ = 2.5–30.0°
*b* = 8.2304 (2) Å	µ = 0.21 mm^−^^1^
*c* = 11.1488 (3) Å	*T* = 100 K
β = 101.630 (2)°	Block, colourless
*V* = 3781.78 (17) Å^3^	0.29 × 0.13 × 0.05 mm
*Z* = 8	

### Data collection

**Table:**

Bruker SMART APEXII CCD diffractometer	3486 independent reflections
Radiation source: fine-focus sealed tube	2496 reflections with *I* > 2σ(*I*)
graphite	*R*_int_ = 0.059
φ and ω scans	θ_max_ = 25.5°, θ_min_ = 2.0°
Absorption correction: multi-scan (*SADABS*; Bruker, 2009)	*h* = −50→48
*T*_min_ = 0.942, *T*_max_ = 0.989	*k* = −9→9
11383 measured reflections	*l* = −13→13

### Refinement

**Table:**

Refinement on *F*^2^	Primary atom site location: structure-invariant direct methods
Least-squares matrix: full	Secondary atom site location: difference Fourier map
*R*[*F*^2^ > 2σ(*F*^2^)] = 0.051	Hydrogen site location: inferred from neighbouring sites
*wR*(*F*^2^) = 0.101	H-atom parameters constrained
*S* = 1.03	*w* = 1/[σ^2^(*F*_o_^2^) + (0.0388*P*)^2^ + 2.5537*P*] where *P* = (*F*_o_^2^ + 2*F*_c_^2^)/3
3486 reflections	(Δ/σ)_max_ < 0.001
272 parameters	Δρ_max_ = 0.38 e Å^−^^3^
0 restraints	Δρ_min_ = −0.40 e Å^−^^3^

### Special details

**Table:**

Experimental. The crystal was placed in the cold stream of an Oxford Cryosystems Cobra open-flow nitrogen cryostat (Cosier & Glazer, 1986) operating at 100.0 (1)K.
Geometry. All esds (except the esd in the dihedral angle between two l.s. planes) are estimated using the full covariance matrix. The cell esds are taken into account individually in the estimation of esds in distances, angles and torsion angles; correlations between esds in cell parameters are only used when they are defined by crystal symmetry. An approximate (isotropic) treatment of cell esds is used for estimating esds involving l.s. planes.
Refinement. Refinement of F^2^ against ALL reflections. The weighted R-factor wR and goodness of fit S are based on F^2^, conventional R-factors R are based on F, with F set to zero for negative F^2^. The threshold expression of F^2^ > 2sigma(F^2^) is used only for calculating R-factors(gt) etc. and is not relevant to the choice of reflections for refinement. R-factors based on F^2^ are statistically about twice as large as those based on F, and R- factors based on ALL data will be even larger.

### Fractional atomic coordinates and isotropic or equivalent isotropic displacement parameters (Å^2^)

**Table:**

	*x*	*y*	*z*	*U*_iso_*/*U*_eq_	
S1	0.220066 (15)	0.57970 (8)	0.11452 (6)	0.01457 (18)	
O1	0.10960 (4)	0.4996 (2)	0.35734 (14)	0.0177 (4)	
O2	0.19581 (4)	−0.0842 (2)	−0.00307 (14)	0.0172 (4)	
O3	0.24309 (4)	0.0525 (2)	0.06238 (15)	0.0184 (4)	
N1	0.16869 (5)	0.7388 (3)	0.34427 (18)	0.0153 (5)	
N2	0.19003 (5)	0.7681 (3)	0.26397 (18)	0.0152 (5)	
N3	0.17980 (5)	0.5056 (3)	0.26683 (17)	0.0129 (5)	
N4	0.17428 (5)	0.3462 (3)	0.22624 (17)	0.0129 (5)	
N5	0.16398 (5)	−0.0737 (3)	0.00868 (18)	0.0167 (5)	
N6	0.16286 (5)	0.0537 (3)	0.07725 (18)	0.0132 (5)	
C1	0.05562 (6)	0.4348 (4)	0.3630 (2)	0.0206 (7)	
H1A	0.0495	0.4827	0.2862	0.025*	
C2	0.03244 (6)	0.3680 (4)	0.4195 (2)	0.0245 (7)	
H2A	0.0108	0.3711	0.3796	0.029*	
C3	0.04051 (6)	0.2958 (4)	0.5348 (2)	0.0204 (7)	
C4	0.07298 (6)	0.2943 (3)	0.5906 (2)	0.0211 (7)	
H4A	0.0790	0.2479	0.6679	0.025*	
C5	0.09682 (6)	0.3592 (3)	0.5357 (2)	0.0187 (6)	
H5A	0.1185	0.3552	0.5753	0.022*	
C6	0.08808 (6)	0.4304 (3)	0.4209 (2)	0.0159 (6)	
C7	0.14297 (6)	0.4960 (4)	0.4192 (2)	0.0163 (6)	
H7A	0.1503	0.3844	0.4318	0.020*	
H7B	0.1454	0.5481	0.4986	0.020*	
C8	0.16269 (6)	0.5830 (3)	0.3431 (2)	0.0135 (6)	
C9	0.19615 (6)	0.6265 (3)	0.2203 (2)	0.0129 (6)	
C10	0.22834 (6)	0.3743 (3)	0.1735 (2)	0.0139 (6)	
H10A	0.2405	0.3153	0.1223	0.017*	
H10B	0.2414	0.3788	0.2558	0.017*	
C11	0.19694 (6)	0.2875 (3)	0.1751 (2)	0.0111 (6)	
C12	0.19158 (6)	0.1326 (3)	0.1140 (2)	0.0117 (6)	
C13	0.21431 (6)	0.0430 (3)	0.0616 (2)	0.0138 (6)	
C14	0.13078 (6)	0.0942 (3)	0.0973 (2)	0.0145 (6)	
C15	0.10783 (6)	0.1424 (3)	−0.0032 (2)	0.0178 (6)	
H15A	0.1131	0.1511	−0.0802	0.021*	
C16	0.07679 (6)	0.1774 (4)	0.0136 (2)	0.0230 (7)	
H16A	0.0608	0.2094	−0.0526	0.028*	
C17	0.06952 (6)	0.1648 (3)	0.1288 (2)	0.0225 (7)	
H17A	0.0487	0.1896	0.1399	0.027*	
C18	0.09303 (6)	0.1153 (3)	0.2280 (2)	0.0210 (7)	
H18A	0.0879	0.1066	0.3050	0.025*	
C19	0.12400 (6)	0.0791 (3)	0.2130 (2)	0.0173 (6)	
H19A	0.1399	0.0453	0.2789	0.021*	
C20	0.01502 (7)	0.2227 (4)	0.5972 (3)	0.0314 (8)	
H20D	0.0252	0.1820	0.6764	0.047*	
H20A	0.0043	0.1352	0.5483	0.047*	
H20B	−0.0006	0.3044	0.6066	0.047*	

### Atomic displacement parameters (Å^2^)

**Table:**

	*U*^11^	*U*^22^	*U*^33^	*U*^12^	*U*^13^	*U*^23^
S1	0.0188 (3)	0.0096 (4)	0.0166 (3)	0.0008 (3)	0.0066 (2)	−0.0007 (3)
O1	0.0168 (9)	0.0222 (12)	0.0141 (9)	−0.0022 (9)	0.0028 (7)	0.0017 (9)
O2	0.0226 (10)	0.0125 (11)	0.0172 (9)	0.0016 (9)	0.0059 (7)	−0.0051 (9)
O3	0.0185 (10)	0.0192 (12)	0.0184 (9)	0.0015 (9)	0.0057 (7)	−0.0013 (9)
N1	0.0197 (12)	0.0147 (14)	0.0119 (11)	0.0013 (11)	0.0039 (9)	−0.0007 (11)
N2	0.0194 (12)	0.0124 (14)	0.0144 (11)	0.0007 (10)	0.0050 (9)	−0.0002 (11)
N3	0.0160 (11)	0.0083 (13)	0.0140 (11)	0.0006 (10)	0.0026 (9)	−0.0036 (11)
N4	0.0200 (11)	0.0073 (13)	0.0116 (10)	0.0006 (10)	0.0033 (9)	0.0009 (11)
N5	0.0208 (12)	0.0139 (14)	0.0156 (11)	0.0019 (11)	0.0044 (9)	−0.0011 (12)
N6	0.0202 (12)	0.0088 (13)	0.0101 (10)	0.0031 (10)	0.0021 (8)	0.0001 (11)
C1	0.0234 (14)	0.0203 (18)	0.0171 (14)	−0.0002 (14)	0.0020 (11)	−0.0008 (14)
C2	0.0171 (14)	0.0258 (19)	0.0298 (16)	−0.0009 (14)	0.0030 (11)	−0.0038 (15)
C3	0.0232 (15)	0.0153 (17)	0.0245 (15)	−0.0003 (13)	0.0093 (12)	−0.0029 (14)
C4	0.0292 (16)	0.0193 (18)	0.0164 (14)	0.0005 (14)	0.0086 (12)	0.0016 (14)
C5	0.0179 (14)	0.0181 (17)	0.0202 (14)	0.0017 (13)	0.0036 (11)	0.0007 (14)
C6	0.0206 (14)	0.0108 (16)	0.0179 (13)	0.0008 (13)	0.0074 (10)	−0.0028 (13)
C7	0.0163 (13)	0.0182 (17)	0.0141 (13)	0.0026 (13)	0.0024 (10)	0.0003 (13)
C8	0.0142 (13)	0.0140 (16)	0.0119 (12)	0.0023 (13)	0.0015 (10)	−0.0012 (14)
C9	0.0162 (13)	0.0105 (16)	0.0112 (13)	−0.0009 (12)	0.0004 (10)	0.0014 (13)
C10	0.0171 (13)	0.0079 (16)	0.0168 (13)	0.0030 (12)	0.0035 (10)	0.0011 (13)
C11	0.0171 (13)	0.0072 (15)	0.0078 (12)	0.0042 (12)	−0.0003 (10)	0.0019 (12)
C12	0.0139 (13)	0.0099 (15)	0.0110 (12)	0.0008 (12)	0.0018 (10)	0.0013 (12)
C13	0.0263 (15)	0.0057 (16)	0.0086 (12)	−0.0009 (13)	0.0016 (10)	0.0015 (12)
C14	0.0152 (13)	0.0099 (16)	0.0181 (13)	−0.0043 (12)	0.0025 (10)	−0.0021 (13)
C15	0.0222 (14)	0.0157 (17)	0.0149 (13)	−0.0029 (13)	0.0026 (11)	0.0012 (13)
C16	0.0210 (15)	0.0209 (19)	0.0256 (15)	−0.0005 (14)	0.0009 (11)	0.0035 (15)
C17	0.0207 (14)	0.0170 (18)	0.0316 (16)	0.0002 (13)	0.0094 (12)	−0.0016 (15)
C18	0.0284 (15)	0.0171 (18)	0.0198 (14)	−0.0048 (14)	0.0105 (12)	−0.0033 (13)
C19	0.0253 (14)	0.0123 (16)	0.0131 (13)	−0.0032 (13)	0.0010 (10)	0.0009 (13)
C20	0.0276 (16)	0.031 (2)	0.0377 (18)	−0.0037 (15)	0.0126 (13)	−0.0001 (17)

### Geometric parameters (Å, °)

**Table:**

S1—C9	1.741 (2)	C4—H4A	0.9300
S1—C10	1.822 (3)	C5—C6	1.387 (3)
O1—C6	1.381 (3)	C5—H5A	0.9300
O1—C7	1.435 (3)	C7—C8	1.485 (3)
O2—N5	1.375 (2)	C7—H7A	0.9700
O2—C13	1.413 (3)	C7—H7B	0.9700
O3—C13	1.212 (3)	C10—C11	1.505 (3)
N1—C8	1.306 (3)	C10—H10A	0.9700
N1—N2	1.411 (3)	C10—H10B	0.9700
N2—C9	1.309 (3)	C11—C12	1.442 (4)
N3—C9	1.369 (3)	C12—C13	1.423 (3)
N3—C8	1.376 (3)	C14—C19	1.382 (3)
N3—N4	1.392 (3)	C14—C15	1.382 (3)
N4—C11	1.299 (3)	C15—C16	1.387 (3)
N5—N6	1.304 (3)	C15—H15A	0.9300
N6—C12	1.360 (3)	C16—C17	1.382 (4)
N6—C14	1.451 (3)	C16—H16A	0.9300
C1—C2	1.378 (4)	C17—C18	1.388 (4)
C1—C6	1.389 (3)	C17—H17A	0.9300
C1—H1A	0.9300	C18—C19	1.380 (3)
C2—C3	1.395 (4)	C18—H18A	0.9300
C2—H2A	0.9300	C19—H19A	0.9300
C3—C4	1.382 (3)	C20—H20D	0.9600
C3—C20	1.516 (4)	C20—H20A	0.9600
C4—C5	1.384 (3)	C20—H20B	0.9600
			
C9—S1—C10	93.16 (12)	N2—C9—S1	129.3 (2)
C6—O1—C7	115.08 (18)	N3—C9—S1	119.9 (2)
N5—O2—C13	110.62 (18)	C11—C10—S1	109.88 (17)
C8—N1—N2	107.9 (2)	C11—C10—H10A	109.7
C9—N2—N1	106.4 (2)	S1—C10—H10A	109.7
C9—N3—C8	105.3 (2)	C11—C10—H10B	109.7
C9—N3—N4	128.70 (19)	S1—C10—H10B	109.7
C8—N3—N4	124.3 (2)	H10A—C10—H10B	108.2
C11—N4—N3	113.8 (2)	N4—C11—C12	118.5 (2)
N6—N5—O2	104.90 (18)	N4—C11—C10	123.5 (2)
N5—N6—C12	115.2 (2)	C12—C11—C10	117.9 (2)
N5—N6—C14	114.8 (2)	N6—C12—C13	105.0 (2)
C12—N6—C14	129.9 (2)	N6—C12—C11	127.6 (2)
C2—C1—C6	119.8 (2)	C13—C12—C11	126.7 (2)
C2—C1—H1A	120.1	O3—C13—O2	119.9 (2)
C6—C1—H1A	120.1	O3—C13—C12	135.8 (2)
C1—C2—C3	121.9 (2)	O2—C13—C12	104.3 (2)
C1—C2—H2A	119.1	C19—C14—C15	122.7 (2)
C3—C2—H2A	119.1	C19—C14—N6	119.8 (2)
C4—C3—C2	117.0 (2)	C15—C14—N6	117.5 (2)
C4—C3—C20	121.1 (2)	C14—C15—C16	118.2 (2)
C2—C3—C20	121.9 (2)	C14—C15—H15A	120.9
C3—C4—C5	122.4 (3)	C16—C15—H15A	120.9
C3—C4—H4A	118.8	C17—C16—C15	120.1 (2)
C5—C4—H4A	118.8	C17—C16—H16A	120.0
C4—C5—C6	119.3 (2)	C15—C16—H16A	120.0
C4—C5—H5A	120.3	C16—C17—C18	120.4 (2)
C6—C5—H5A	120.3	C16—C17—H17A	119.8
O1—C6—C5	124.7 (2)	C18—C17—H17A	119.8
O1—C6—C1	115.8 (2)	C19—C18—C17	120.4 (2)
C5—C6—C1	119.5 (2)	C19—C18—H18A	119.8
O1—C7—C8	108.69 (19)	C17—C18—H18A	119.8
O1—C7—H7A	110.0	C18—C19—C14	118.1 (2)
C8—C7—H7A	110.0	C18—C19—H19A	120.9
O1—C7—H7B	110.0	C14—C19—H19A	120.9
C8—C7—H7B	110.0	C3—C20—H20D	109.5
H7A—C7—H7B	108.3	C3—C20—H20A	109.5
N1—C8—N3	109.7 (2)	H20D—C20—H20A	109.5
N1—C8—C7	126.6 (2)	C3—C20—H20B	109.5
N3—C8—C7	123.5 (2)	H20D—C20—H20B	109.5
N2—C9—N3	110.8 (2)	H20A—C20—H20B	109.5
			
C8—N1—N2—C9	−1.1 (3)	C10—S1—C9—N2	154.0 (2)
C9—N3—N4—C11	30.0 (3)	C10—S1—C9—N3	−28.0 (2)
C8—N3—N4—C11	−167.4 (2)	C9—S1—C10—C11	54.13 (18)
C13—O2—N5—N6	0.0 (2)	N3—N4—C11—C12	−171.17 (19)
O2—N5—N6—C12	0.0 (3)	N3—N4—C11—C10	7.4 (3)
O2—N5—N6—C14	176.97 (18)	S1—C10—C11—N4	−52.4 (3)
C6—C1—C2—C3	0.4 (4)	S1—C10—C11—C12	126.2 (2)
C1—C2—C3—C4	0.1 (4)	N5—N6—C12—C13	0.0 (3)
C1—C2—C3—C20	179.9 (3)	C14—N6—C12—C13	−176.4 (2)
C2—C3—C4—C5	−0.6 (4)	N5—N6—C12—C11	170.7 (2)
C20—C3—C4—C5	179.6 (3)	C14—N6—C12—C11	−5.7 (4)
C3—C4—C5—C6	0.7 (4)	N4—C11—C12—N6	16.6 (4)
C7—O1—C6—C5	−0.8 (4)	C10—C11—C12—N6	−162.0 (2)
C7—O1—C6—C1	179.2 (2)	N4—C11—C12—C13	−174.6 (2)
C4—C5—C6—O1	179.7 (3)	C10—C11—C12—C13	6.8 (4)
C4—C5—C6—C1	−0.2 (4)	N5—O2—C13—O3	179.4 (2)
C2—C1—C6—O1	179.8 (2)	N5—O2—C13—C12	0.0 (2)
C2—C1—C6—C5	−0.3 (4)	N6—C12—C13—O3	−179.3 (3)
C6—O1—C7—C8	−176.7 (2)	C11—C12—C13—O3	9.9 (5)
N2—N1—C8—N3	1.3 (3)	N6—C12—C13—O2	0.0 (2)
N2—N1—C8—C7	176.2 (2)	C11—C12—C13—O2	−170.8 (2)
C9—N3—C8—N1	−1.1 (3)	N5—N6—C14—C19	112.8 (3)
N4—N3—C8—N1	−167.0 (2)	C12—N6—C14—C19	−70.9 (4)
C9—N3—C8—C7	−176.1 (2)	N5—N6—C14—C15	−65.3 (3)
N4—N3—C8—C7	18.0 (3)	C12—N6—C14—C15	111.1 (3)
O1—C7—C8—N1	87.1 (3)	C19—C14—C15—C16	0.3 (4)
O1—C7—C8—N3	−98.7 (3)	N6—C14—C15—C16	178.3 (2)
N1—N2—C9—N3	0.5 (3)	C14—C15—C16—C17	0.4 (4)
N1—N2—C9—S1	178.59 (18)	C15—C16—C17—C18	−0.7 (4)
C8—N3—C9—N2	0.3 (3)	C16—C17—C18—C19	0.4 (4)
N4—N3—C9—N2	165.4 (2)	C17—C18—C19—C14	0.3 (4)
C8—N3—C9—S1	−178.00 (17)	C15—C14—C19—C18	−0.7 (4)
N4—N3—C9—S1	−12.9 (3)	N6—C14—C19—C18	−178.6 (2)

### Hydrogen-bond geometry (Å, °)

**Table:**

*D*—H···*A*	*D*—H	H···*A*	*D*···*A*	*D*—H···*A*
C10—H10A···O3	0.97	2.27	3.041 (3)	135
C10—H10A···O3^i^	0.97	2.54	3.162 (3)	122
C10—H10B···O3^ii^	0.97	2.46	3.292 (3)	144
C19—H19A···N5^iii^	0.93	2.57	3.386 (3)	147

Symmetry codes: (i) −*x*+1/2, −*y*+1/2, −*z*; (ii) −*x*+1/2, *y*+1/2, −*z*+1/2; (iii) *x*, −*y*, *z*+1/2.
